# Role of dendritic cells and B cells in the skin of imiquimod (IMQ)-induced psoriasis-like mouse model

**DOI:** 10.7717/peerj.20974

**Published:** 2026-03-30

**Authors:** Aina Akmal Mohd Noor, Lidawani Lambuk, Farizan Ahmad, Maryam Azlan, Norhanani Mohd Redzwan

**Affiliations:** 1 Department of Immunology, School of Medical Sciences, Universiti Sains Malaysia, Kubang Kerian, Kelantan, Malaysia; 2 Optometry and Vision Science Programme, Center for Community Health Studies (ReaCH), Faculty of Health Sciences, Universiti Kebangsaan Malaysia, Kuala Lumpur, Kuala Lumpur, Malaysia; 3 Department of Neurosciences, School of Medical Sciences, Universiti Sains Malaysia, Kubang Kerian, Kelantan, Malaysia; 4 School of Health Sciences, Universiti Sains Malaysia, Kubang Kerian, Kelantan, Malaysia

**Keywords:** Psoriasis, Skin, Dendritic cell, B cell, Imiquimod, Mouse model

## Abstract

**Background:**

Psoriasis is a chronic inflammatory skin disease traditionally characterized by T cell-mediated immune responses. However, dendritic cells (DCs) and B cells also contribute to immune regulation in psoriasis. Their role in disease progression remains underexplored.

**Methods:**

This study utilized an imiquimod (IMQ)-induced psoriasis-like mouse model to investigate DC and B cell dynamics at different stages of disease progression (day 3, day 5 and day 7). Skin lesions were evaluated using the Psoriasis Area and Severity Index (PASI) and histological analysis. Flow cytometry was performed to assess the expression of CD11c^+^MHCII^+^ DCs and CD19^+^CD38^+^ B cells, while gene expression analysis using reverse transcriptase polymerase chain reaction (RT-PCR) of *CD11c, H2-Aa, B cell activating factor (BAFF), interleukin (IL)-10, IL-6* and *CXCR5* was conducted to elucidate immune modulation within the psoriasis skin microenvironment.

**Results:**

IMQ application induced progressive skin thickening, erythema and scaling, with PASI scores significantly increased at day 7 compared to controls (*p* = 0.002), reflecting pronounced inflammation. Histological analysis revealed epidermal hyperplasia, hyperkeratosis and dermal immune cell infiltration. Skin CD11c^+^MHCII^+^ DCs were significantly elevated at day 3 (*p* < 0.01), corresponding with upregulation of *CD11c* and *H2-Aa* genes, while CD19^+^CD38^+^ B cells expanded at later stages, peaking at day 7 (*p* < 0.0001), associated with increased *BAFF*, *IL-10*, *IL-6* and *CXCR5* expression. A reduction in circulating B cells was observed alongside increased skin infiltration, suggesting a possible redistribution associated with inflammation.

**Conclusion:**

Early DC activation corresponded with the initiation phase of inflammation, while the later expansion of B cells appeared to coincide with sustained inflammatory activity and disease progression. The coordinated presence of DCs and B cells in the skin microenvironment may influence disease severity and pathogenesis, which could provide fundamental insights for future investigations into potential therapeutic strategies.

## Introduction

Psoriasis is a chronic inflammatory skin disease characterized by T cell-driven immune responses and inflammation (Zhang et al., 2023). Current therapeutic strategies, particularly biologics targeting T cell-mediated pathways such as tumor necrosis factor (TNF)-α inhibitors, interleukin (IL)-12/23 inhibitors and IL-17 inhibitors, have effectively managed psoriasis inflammation (Torres et al., 2021; Lin et al., 2024; Wride et al., 2024). However, despite the success of these treatments for many patients, there remain possible challenges, such as incomplete responses, recurrence of symptoms and the need for lifelong therapy (Iannone et al., 2020; Sutaria & Au, 2021; Al-Janabi & Yiu, 2022). These limitations have raised questions about whether a more comprehensive understanding of the immune mechanisms driving psoriasis could open new avenues for therapy.

While traditionally recognized as a T cell-mediated disorder, emerging evidence suggests that additional immune cells contribute to its pathogenesis. The immune landscape in psoriasis extends beyond T cell-driven inflammation, with dendritic cells (DCs) and B cells playing fundamental roles in modulating immune responses. DCs, pivotal in initiating and regulating immune responses through antigen presentation and T cell activation, have been shown to contribute to inflammation in various autoimmune diseases, including psoriasis (Torres-Ruiz & Shoenfeld, 2019). Similarly, B cells, traditionally known for their role in antibody production, are emerging as critical modulators of inflammation in psoriasis (Alrefai et al., 2016). They have been implicated in producing pro-inflammatory cytokines (De Gruijter, Jebson & Rosser, 2022) and regulating the activation of T cells and other immune responses (Fetter et al., 2020; Mohd Noor, Azlan & Mohd Redzwan, 2022; Sun et al., 2024a).

Reviews continue to emphasize that B cells have thus far largely been neglected in psoriasis research (Grän et al., 2020). Recent work using single-cell sequencing has begun to characterize DC subset heterogeneity in psoriatic lesions, identifying novel DC-associated biomarkers such as Fatty Acid Binding Protein 5 (FABP5) and killer cell lectin-like receptor B1 (KLRB1), yet the precise functional contributions of these subsets to disease pathogenesis remain unclear (Ma et al., 2024). Similarly, analyses of peripheral blood DC subsets in severe psoriasis have demonstrated altered frequencies of cDC2 and DC3 during biologic therapy, but the mechanisms by which these subsets drive or sustain inflammation in skin are not fully defined (Petrovic et al., 2023). Broader reviews also highlight that while IL-23-producing DCs are recognized as pivotal drivers of the psoriatic loop, the interplay between DCs, B cells and other immune cells remains insufficiently mapped (Sieminska, Pieniawska & Grzywa, 2024). In addition, the skin immune microenvironment has been increasingly studied using systems immunology approaches, but the contribution of DC–B cell interactions is still poorly defined compared to T cell pathways (Yao et al., 2025). Collectively, these findings underscore that the functions of DCs and B cells in psoriasis are underexplored relative to T cells and require deeper investigation.

Targeting DCs and B cells has shown therapeutic potential in other autoimmune diseases, suggesting their relevance in psoriasis. Biologics like abatacept, which block CD80/CD86 interactions on DCs to reduce T cell activation, have been effective in rheumatoid arthritis (Ahamada & Wu, 2023). Furthermore, iscalimab, an anti-CD40 antibody, inhibits the CD40-CD40L interaction on DCs, limiting T cell activation in conditions such as Sjögren’s syndrome and renal transplantation (Nashan et al., 2018; El Jamal & Shibli, 2024). Additionally, targeting B cells has also been beneficial, as seen with belimumab, a B cell activating factor (BAFF) inhibitor (Álvarez et al., 2023). This inhibitor reduces BAFF-mediated B cell survival and autoantibody production in systemic lupus erythematosus. Novel strategies, such as using polylactic acid nanoparticles to deliver Janus kinase (JAK) inhibitors specifically to B cells, have further demonstrated efficacy in suppressing B cell activation and inflammatory cytokine production during SLE flares (Moura & Fonseca, 2021; Álvarez et al., 2023). Given the growing recognition of their roles in immune regulation and inflammation, there is a compelling reason to explore DCs and B cells as potential therapeutic targets in psoriasis. The success of immunomodulatory therapies in other autoimmune diseases warrants the need to investigate their contributions to psoriasis pathogenesis, potentially guiding future therapeutic strategies.

To address this gap, the current study employs an imiquimod (IMQ)-induced mouse model and adopts a time-based approach to capture the involvement of DCs and B cells during disease progression. By analyzing DCs and B cells at strategic time points, the study aims to elucidate their roles in psoriasis-like inflammation. This approach provides insights into the stage-specific contributions of DCs and B cells to psoriasis pathogenesis, particularly their roles in immune dysregulation, cytokine production and antigen presentation. While T cell-focused therapies often fail to achieve long-term remission, understanding the immunopathological contributions of DCs and B cells may pave the way for more effective treatment strategies.

## Materials & Methods

### Induction of psoriasis-like inflammation using animal model

A total of 36 male BALB/c mice (6–8 weeks old, weighing 18–23 g) were purchased from the Animal Research and Service Centre at Universiti Sains Malaysia with the reference approved animal ethical number: USM/IACUC/2019/(120)(1029). The number of animals used was determined based on previous studies to ensure statistical robustness while minimizing animal usage. Only male mice were used to reduce hormonal variability that can influence immune responses and psoriasis-like skin inflammation to ensure more consistent and reproducible outcomes (Wu et al., 2022). The mice were housed under standard laboratory conditions, maintained at a temperature of 22 ± 2 °C with a 12-hour light/dark cycle and provided *ad libitum* access to food and water. Environmental enrichment was provided using corncob bedding as nesting material and shelter. The mice were randomly assigned to two main groups: control and IMQ-induced. The mice were further subdivided within each group based on the analysis time points on day 3, day 5 and day 7, with six mice in each subgroup (*n* = 6). The sample size was determined using PS Software (version 3.1.2) based on previous IMQ-induced psoriasis-like studies (Van Der Fits et al., 2009; Swindell et al., 2017). The calculation parameters included α (type 1 error probability) = 0.05, *β* (power of study) = 0.9, *δ* (difference in means for the selected parameter between IMQ-induced and control mice) = 1.090 and *σ* (within-group standard deviation) = 0.455, with a 1:1 ratio of control to experimental groups. The minimum sample size required per group was five mice, with an additional 20% dropout consideration (one mouse per group), resulting in a final sample size of six mice per group. A 3 × three cm area on the upper back was shaved to induce psoriasis-like lesions and depilation was achieved using Veet^®^ cream (VEET, Nutley, NJ, USA). Anesthesia was maintained with 4% isoflurane inhalation during the shaving procedure. For psoriasis-like induction, 62.5 mg Aldara cream containing 5% imiquimod (Aldara, iNova Pharmaceuticals, Singapore) was applied to the shaved area, while the control group received Vaseline^®^ (Unilever, Hoboken, NJ, USA) as a standard emollient following established protocols and a pilot trial (Noor et al., 2021). Psoriasis severity was assessed by measuring skin inflammation, thickness and PASI scoring. Criteria for humane endpoint euthanasia included severe ulceration, weight loss exceeding 20%, or signs of distress, though no early euthanasia was required. At the endpoint, skin samples were collected for analysis. Mice were euthanized using an intraperitoneal injection of sodium pentobarbital (60 mg/kg) (Alfasan Woerden-Holland Woerden, Netherlands). All animals were euthanized after the experiment; no surviving animals remained.

### Evaluation of psoriasis-like skin severity

Skinfold thickness was measured at designated time points using vernier calipers (Mitutoyo, Japan) under the supervision of a veterinary physician. Further psoriasis-like lesion severity was assessed using a modified Psoriasis Area and Severity Index (PASI) score, as adapted from Van Der Fits et al. (2009). Mice were randomly assigned to the control or IMQ-treated group (*n* = 6 per group) using a simple randomization method, where group allocation was determined by drawing coded labels from sealed envelopes prepared by an independent individual. Allocation was concealed by labelling the cages with non-identifiable codes prepared by separate personnel not involved in data collection. During the evaluation phase, three independent veterinary physicians, blinded to the treatment groups, scored erythema, thickness and scaling daily on a scale from 0 to 4 (0 = none, 1 = slight, 2 = moderate, 3 = marked, 4 = very marked). Inter-rater reliability was assessed to ensure consistency and minimize observer-related variability in PASI scoring. The cumulative score, ranging from 0 to 12, represented the overall severity of skin inflammation.

### Histology procedures

Skin samples were fixed in 10% neutral buffered formalin (Leica Biosystems, Germany), dehydrated in graded ethanol (HmbG, Malaysia), cleared with xylene (Merck, Germany) and embedded in paraffin (Leica Biosystems, Wetzlar, Germany). Sections were prepared using a microtome and mounted on slides. Hematoxylin and eosin (H&E) staining involved deparaffinization, staining with hematoxylin and eosin (Merck, Darmstadt) and clearing with ethanol and xylene before mounting with DPX (R&M Marketing). For Masson’s Trichrome staining, slides were additionally immersed in Bouin’s solution (Sigma-Aldrich, St. Louis, MO, USA) before staining with Weigert’s iron hematoxylin (Sigma-Aldrich), Biebrich-Scarlet acid fuchsin (Biognost, Croatia), phosphomolybdic acid (Sigma-Aldrich, USA) and light green (Histoline, Milan, Italy). Slides were dehydrated and mounted for microscopic evaluation using Olympus cellSens standard software.

### Flow cytometry

Single-cell suspensions from the skin were prepared with modifications from Lou, Sun & Wang (2020). Harvested skin was washed in pre-chilled Hanks’ balanced salt solution (Sigma-Aldrich, USA) and the epidermis was removed following digestion in Dispase^®^ II protease solution (Sigma-Aldrich). The dermis was minced and incubated in a dissociation buffer containing DNase I Grade II and *Collagenase P* (Roche, Basel, Switzerland), then further mixed and incubated. The suspension was filtered through a 70 µm strainer (Biologix, Chemopharm, Malaysia) using complete Dulbecco’s Modified Eagle Medium (Cytiva Life Sciences, Wilmington, DE, USA) with 10% fetal bovine serum (Sigma-Aldrich). DCs were stained with MHCII-PE and CD11c-PE-Cy-7, while B cells were stained with B220-APC, CD38-FITC and CD19-APC (Thermo Fisher Scientific, Waltham, MA, USA). Samples were analyzed using a FACSCanto II flow cytometer (BD Biosciences, Singapore) and FlowJo^®^ Version 10.8.1.

### Reverse transcriptase polymerase chain reaction (RT-PCR)

RNA extraction from skin samples was performed using the RNeasy Fibrous Tissue Mini Kit (Qiagen). Skin (25–30 mg) was homogenized in RLT buffer with proteinase K, incubated and centrifuged. The supernatant was mixed with ethanol, loaded onto the RNeasy column and processed according to the manufacturer’s protocol, including DNase I treatment. RNA was eluted with RNase-free water. cDNA synthesis was conducted using the Quantinova Reverse Transcription Kit (Qiagen), which included genomic DNA removal. Quantitative PCR was performed in triplicate with a 5-fold dilution series to determine primer efficiency. To ensure reliable normalization, both *GAPDH* and *ACTB* were assessed as reference genes. Their expression stability across experimental groups was evaluated using comparative CT variation, where no significant fluctuations were detected. *GAPDH* showed the lowest variability and was therefore considered the most stable reference, while *ACTB* was included for validation. Gene expression was normalized to these reference genes, analysed using the 2^−ΔΔCT^ method and acquired with CFX Opus 96 Real-Time PCR (Bio-Rad Laboratories, Inc., Hercules, CA, USA) and CFX Maestro Version 2.2 (Bio-Rad Laboratories, Inc.). Primer sequences (5′→3′) are listed in Table 1.

**Table 1:** Primer sequences and annealing temperatures for quantitative gene expression analysis. Forward (F) and reverse (R) primer sequences used for quantitative gene expression analysis, along with their respective accession numbers and annealing temperatures.

Gene	Accession number	Sequence (5′→ 3′)	Annealing temperature (°C)
*GAPDH*	NM_001411843.1	F: ATG TGT CCG TCG TGG ATC TGA C	60.8
		R: AGA CAA CCT GGT CCT CAG TGT AG	
*ACTB*	NM_007393.5	F: CGT TGA CAT CCG TAA AGA CCT C	62.1
		R: ACA GAG TAC TTG CGC TCA GGA G	
*CD11c*	NM_001363985.1	F: ACA CAG TGT GCT CCA GTA TGA	60.8
		R: GCC CAG GGA TAT GTT CAC AGC	
*H2-Aa*	NM_010378.3	F: CTG TGA TCA ACA TCA CAT GGC	54.4
		R: TTG TGG AAG GAA TAG TCA CGG	
*BAFF*	NM_001425009.1	F: TTC CAT GGC TTC TCA GCT TT	61.6
		R: CGT CCC CAA AGA CGT GTA CT	
*IL-10*	NM_010548.2	F: CAC AAA GCA GCC TTG CAG AA	62.1
		R: AGA GCA GGC AGC ATA GCA GTG	
*IL-6*	NM_001314054.1	F: CCA CTT CAC AAG TCG GAG GCT TA	61.2
		R: GCA AGT GCA TCA TCG TTG TTC ATA C	
*CXCR5*	NM_007551.3	F: ATC GTC CAT GCT GTT CAC GCC T	60.8
		R: CAA CCT TGG CAA AGA GGA GTT CC	

**Notes.**

F: forward, R: reverse.

### Statistical analysis

Statistical analyses were conducted using the Student’s *t*-test for parametric data and the Mann–Whitney *U* test for non-parametric data. Comparisons between control and IMQ-treated groups were performed independently at each time point, as separate groups of mice were used for day 3, 5, and 7 measurements. Results are presented as mean ± SD and median difference for all comparisons, with 95% confidence intervals (CI) reported when applicable, and fold change indicated for gene expression analyses. All *p*-values which are < 0.05 considered statistically significant. GraphPad Prism version 9.0.0 software was used for the analyses, with significance indicated as *p < 0.05, **p < 0.01, ***p < 0.001 and ****p < 0.0001.

## Results

### Pathological progression of IMQ-induced psoriasis: macroscopic and histological perspectives

**Figure 1:**
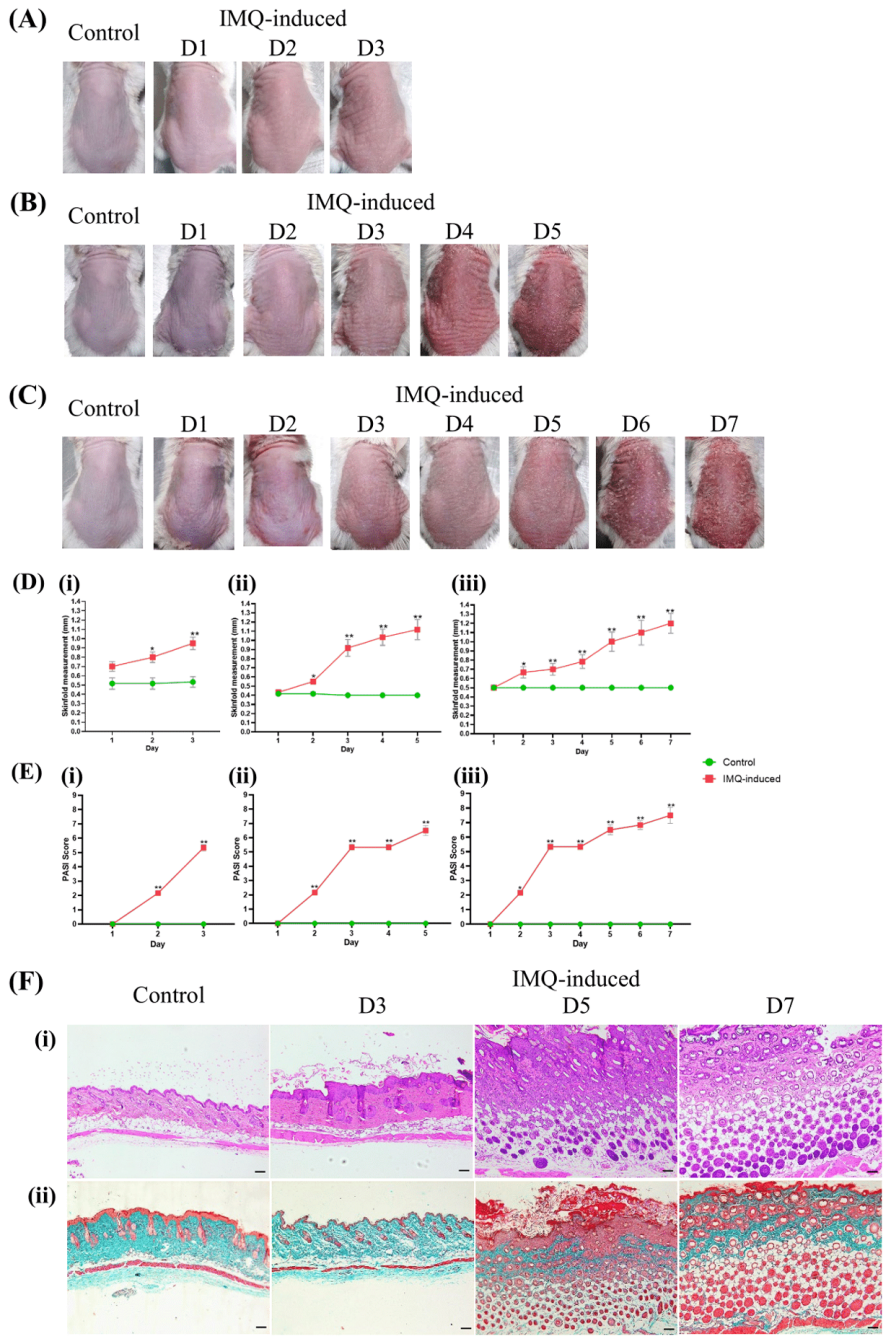
Pathological progression of IMQ-induced psoriasis: macroscopic and histological perspectives. Representative images of back skin appearance in the IMQ-induced psoriasis-like mouse model for control and IMQ-treated groups at (A) days 1–3, (B) days 1–5 and (C) days 1–7. Skin lesions in the IMQ-induced group exhibit progressive erythema, scaling and thickening, while the control group remains unchanged. (D) Skinfold thickness measurements in control (green) and IMQ-induced (red) groups over (i) 3, (ii) 5 and (iii) 7 days, showing a progressive increase in IMQ-induced mice. (E) Cumulative PASI scores over (i) 3, (ii) 5 and (iii) 7 days in IMQ-induced groups, incorporating erythema, scaling and thickening parameters. Each time point represents an independent data set collected on that specific day, with measurements not cumulative or continuous across days. Data (*n* = 6 per group) are presented as mean ± SD (*p < 0.05, **p < 0.01). (F) Histological analysis of skin sections: (i) H&E staining reveals epidermal thickening, hyperkeratosis, psoriasiform hyperplasia and increased capillary density in the IMQ-induced group. (ii) Masson’s Trichrome staining shows red-stained scabs, detached keratinocytes, a thickened dermis with reduced collagen density and prominent blood capillaries. The hypodermis exhibits an increased number of hair follicles. Original magnification, ×10; scale bar, 200 µm.

Daily monitoring of the back skin in IMQ-induced and control groups revealed distinct visual changes corresponding to psoriasis-like characterizations. The back skin of IMQ-induced mice showed progressive development of erythema, scaling and thickening, which became increasingly pronounced and peaked on day 7, whereas the control group exhibited no visible changes (Figs. 1A–1C). Skinfold thickness (Fig. 1D) in IMQ-induced mice was significantly increased compared to controls on (i) day 3 (median difference = 0.50, *p* = 0.002), (ii) day 5 (median difference = 0.60, *p* = 0.002) and (iii) day 7 (median difference = 0.75, *p* = 0.002), as determined by the Mann–Whitney *U* test. Additionally, PASI scores (Fig. 1E) exhibited a clear upward trend, reflecting the severity of skin inflammation and epidermal alterations as psoriasis progressed. PASI scores were significantly increased in IMQ-induced mice compared to controls on (i) day 3 (median difference = 5.00, *p* = 0.002), (ii) day 5 (median difference = 6.00, *p* = 0.002) and (iii) day 7 (median difference = 7.50, *p* = 0.002), as determined by Student’s *t*-test. In contrast, the control group remained stable with negligible PASI scores, indicating the absence of psoriasis-like symptoms. A summary of all mean ± SD values, median differences, 95% CI, and *p* values for each parameter is available in Table S1.

Histological analysis (Fig. 1F) further confirmed these macroscopic observations. Representative H&E-stained sections (Fig. 1Fi) showed progressive epidermal thickening, hyperkeratosis, psoriasiform hyperplasia, increased capillary density and inflammatory infiltration in IMQ-induced mice, intensifying from day 3 to day 7. Masson’s trichrome staining (Fig. 1Fii) revealed red-stained scabs, detached keratinocytes, a thickened dermis with reduced collagen density and prominent blood capillaries. The hypodermis exhibits an increased number of hair follicles. In contrast, the control group displayed normal epidermal thickness with minimal inflammatory infiltration and no evident collagen remodelling. These findings collectively demonstrate the progressive nature of IMQ-induced psoriasis-like skin alterations.

### Skin DC and B cell dynamics with progressive gene expression changes in IMQ-induced psoriasis

**Figure 2:**
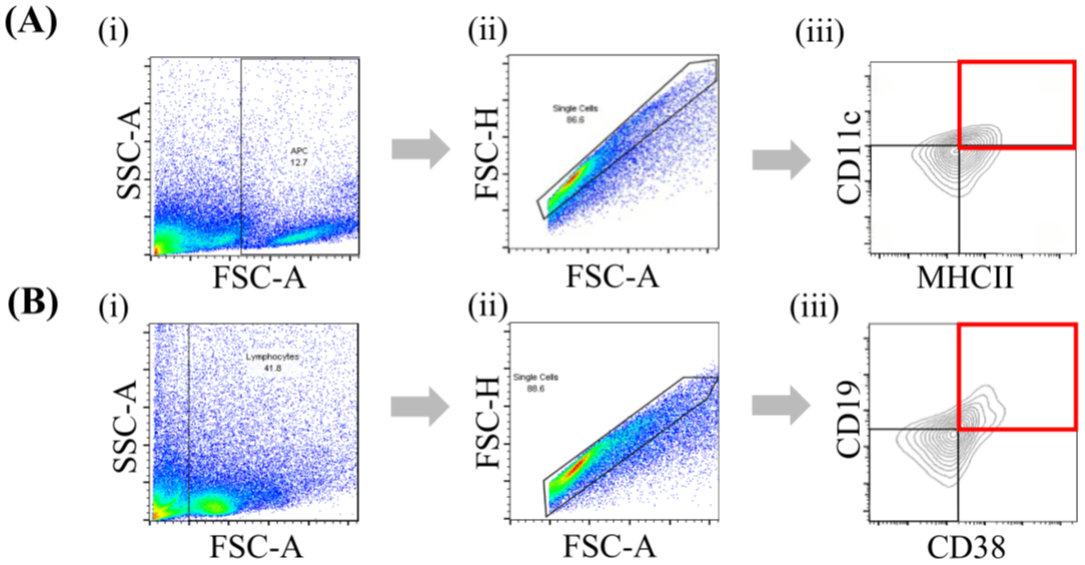
Gating strategy for DCs and B cells in skin samples using control subjects. Cells were first gated based on size and granularity using SSC-A *vs.* FSC-A, followed by singlet exclusion using FSC-H *vs.* FSC-A. (A) DCs were identified as CD11c^+^MHCII^+^ and (B) B cells were defined as CD19^+^CD38^+^, with the gated populations highlighted in red.

**Figure 3:**
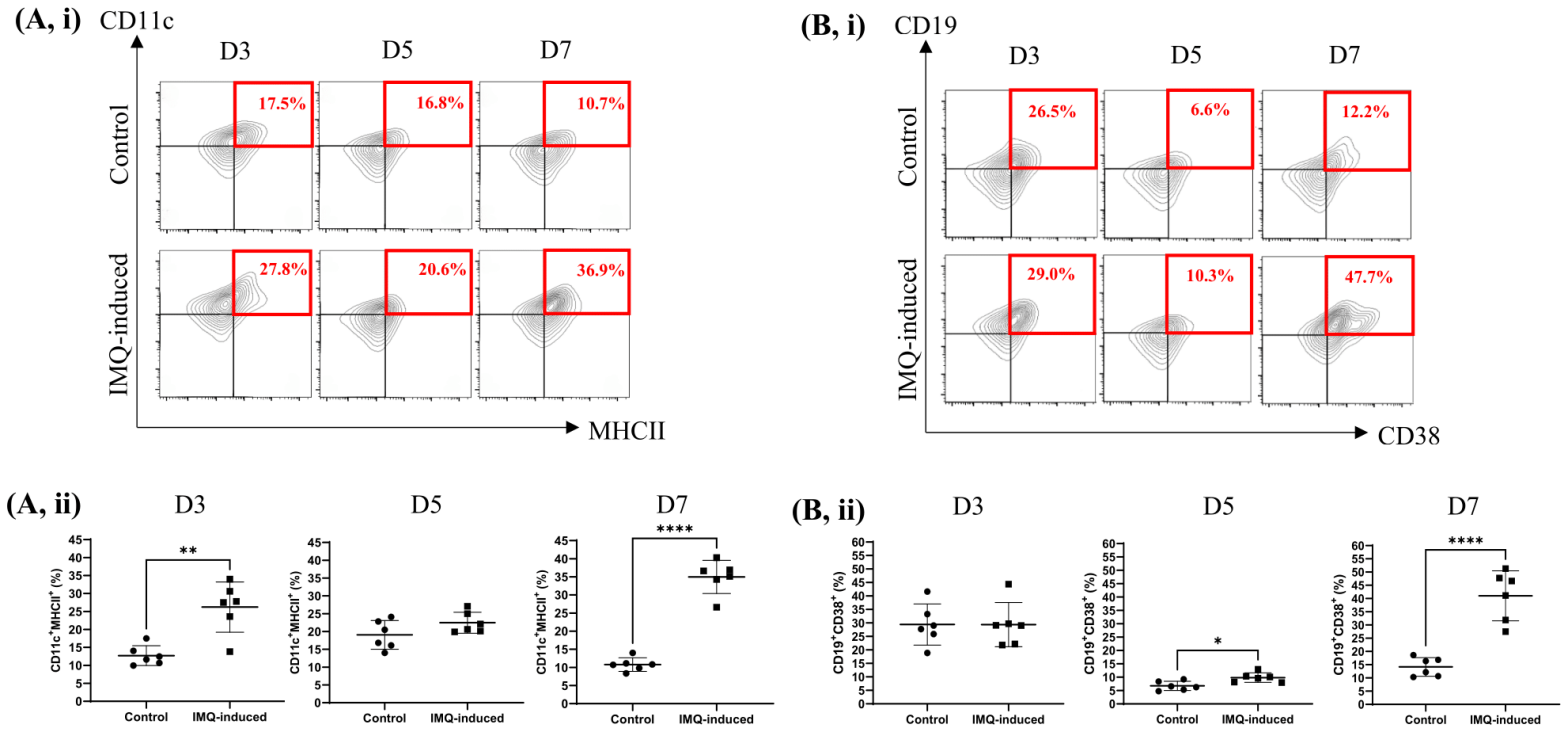
Progressive changes in DCs and B cell populations in the skin following IMQ induction. (A, i) Representative contour plots of CD11c^+^MHCII^+^ DCs at day 3, day 5, and day 7 in control and IMQ-induced groups. (ii) Quantitative analysis reveals significant increases in the IMQ-induced group compared to the control group on day 3 (*p* < 0.01) and day 7 (*p* < 0.0001). (B, i) Representative contour plots of CD19^+^CD38^+^ B cells at day 3, day 5, and day 7 in control and IMQ-induced groups. (ii) Quantitative analysis shows no significant difference on day 3 (*p* = 0.9943), with significant increases on day 5 (*p* < 0.05) and day 7 (*p* < 0.0001) in the IMQ-induced group compared to the control. D3 = day 3, D5 = day 5, D7 = day 7, data (*n* = 6 for each group) are expressed as mean ± SD (*p < 0.05, **p < 0.01, ****p < 0.0001).

Skin DCs and B cells were identified using sequential gating based on size, singlet exclusion and marker expression. Cells were first gated based on forward scatter (FSC-A) and side scatter (SSC-A) to exclude debris, followed by doublet discrimination using FSC-A *versus* FSC-H. Viable singlets were subsequently analysed for marker expression. Double-positive CD11c^+^ and MHCII^+^ cells were identified as DCs, while double-positive CD19^+^ and CD38^+^ cells were identified as B cells, as illustrated in Figs. 2A and 2B, respectively. The flow cytometry analysis revealed dynamic changes in skin DCs and B cell populations following IMQ induction. In Fig. 3A, CD11c^+^MHCII^+^ DCs increased significantly in the IMQ-induced group compared to the control, with a marked rise on day 3 (median difference = 26.83, 95% CI [18.97–31.75], *p* < 0.01) and a peak on day 7 (median difference = 25.10, 95% CI [19.67–28.68], *p* < 0.0001). In contrast, the control group showed a lower and more stable DC population over time. Day 5 showed a transient plateau, with only a slight difference of 2.70 compared to controls (median difference = 2.70, 95% CI [−1.13 to 7.96], *p* = 0.1250), before the subsequent surge. In Fig. 3B, CD19^+^CD38^+^ B cells remained similar between groups on day 3 (median difference = 0.70, 95% CI [−10.20 to 10.13], *p* = 0.9943) but exhibited a delayed response, showing a moderate increase on day 5 (median difference = 3.45, 95% CI [0.83–5.37], *p* = 0.0124) and a substantial expansion by day 7 in the IMQ-induced group (median difference = 29.55, 95% CI [17.66–36.04], *p* < 0.0001). This progressive shift suggests an evolving immune response, with DC activation preceding the pronounced B cell accumulation.

**Figure 4:**
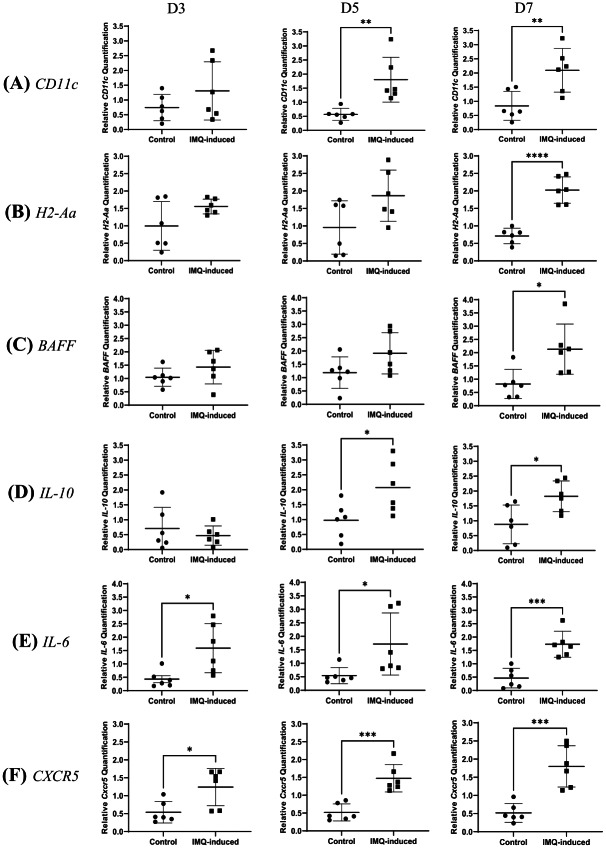
Relative gene expression quantification levels . (A) *CD11c*, (B) *H2-Aa*, (C) *BAFF*, (D) *IL-10*, (E) *IL-6* and (F) *CXCR5* expressions in skin samples from control and IMQ-induced groups at day 3, day 5 and day 7. IMQ-induced groups show significantly elevated expressions compared to controls at all time points. Data (*n* = 6 for each group) represent mean ± SD. *p < 0.05, **p < 0.01, ***p < 0.001, ****p < 0.0001.

To further investigate immune alterations in the IMQ-induced model, gene expression analysis was performed in the skin to examine molecular changes in relation to cellular changes. Gene expression analysis in skin tissue revealed progressive changes in the IMQ-induced group compared to the control (Fig. 4). Figure 4A shows that *CD11c* expression remained unchanged at the early phase on day 3 (1.76-fold, median difference = 0.27, 95% CI [−0.42 to 1.55], *p* = 0.2287) but was significantly upregulated from day 5 (3.18-fold, median difference = 0.88, 95% CI [0.48–1.99], *p* = 0.044) to day 7 (2.51-fold; median difference = 1.53, 95% CI [0.42–2.10], *p* = 0.0075), aligning with the increase in CD11c^+^MHCII^+^ DCs observed by flow cytometry. Furthermore, Fig. 4B shows that *H2-Aa* expression remained stable during the initial phase (day 3: 1.56-fold, median difference = 0.73, 95% CI [−0.10–1.23], *p* = 0.0895; day 5: 1.86-fold, median difference = 0.67, 95% CI [0.05–1.87], *p* = 0.0616) but showed a significant elevation at later stages on day 7 (2.02-fold, median difference = 1.25, 95% CI [0.91–1.71], *p* < 0.0001), suggesting its involvement in disease progression. Similarly, Fig. 4C demonstrates that *BAFF* expression did not change significantly in the early phases (day 3: 1.36-fold, median difference = 0.78, 95% CI [−0.27 to 1.03], *p* = 0.2210; day 5: 1.61-fold, median difference = 0.48, 95% CI [−0.16 to 1.61], *p* = 0.0980) but was markedly increased by day 7 (2.60-fold, median difference = 1.31, 95% CI [0.32–2.31], *p* = 0.0150), aligning with the substantial expansion of CD19^+^CD38^+^ B cells, further supporting a delayed but progressive B cell response. Interestingly, Fig. 4D shows *IL-10* expression exhibited an early increase on day 3 (2.07-fold, median difference = 0.92, 95% CI [0.19–1.10], *p* = 0.019), which continued to day 5 (2.12-fold, median difference = 0.84, 95% CI [0.14–2.05], *p* = 0.0280), but by day 7, it declined and was not significantly different from the control (0.66-fold,median difference = −0.0009, 95% CI [−0.95 to 0.47], *p* = 0.4710). Meanwhile, Fig. 4E demonstrates that *IL-6* remained consistently elevated throughout (day 3: 2.94-fold, median difference = 1.14, 95% CI [0.27–2.05], *p* = 0.0155; day 5: 2.81-fold, median difference = 0.69, *p* = 0.0152; day 7: 3.74-fold, median difference = 1.28, 95% CI [0.71–1.83], *p* = 0.0005), reflecting a sustained inflammatory response. Figure 4F shows that *CXCR5* expression increased progressively, reaching significance on day 3 (2.30-fold, median difference = 1.08, *p* = 0.0260), rising further on day 5 (2.85-fold, median difference = 0.911, 95% CI [0.55–1.37], *p* = 0.0004) and showing strong upregulation by day 7 (3.47-fold, median difference = 1.351, 95% CI [0.71–1.85], *p* = 0.0005). This upward trend paralleled the observed increase in skin B cell populations. A summary of all mean ± SD, median differences, 95% CI, and *p* values for each parameter calculated by either Student’s *t*-test or Mann–Whitney *U* test for each parameter is available in Table S1. Overall, these findings indicate a sequential immune response in IMQ-induced skin inflammation, where early DC activation precedes a delayed but substantial B cell expansion, accompanied by progressive changes in key immune-related gene expressions.

## Discussion

Psoriasis is a chronic inflammatory skin disorder characterized by dysregulated immune responses involving DCs, B cells and T cells. The IMQ-induced psoriasis-like mouse model is widely used to study psoriasis pathogenesis due to its ability to replicate key features of human psoriasis, including epidermal hyperplasia, immune infiltration and cytokine dysregulation (Badanthadka & D’Souza, 2020; Yin et al., 2024). Topical application of 62.5 mg IMQ cream, the optimal dose for inducing psoriasis-like lesions, activates toll-like receptor (TLR)7 on DCs, triggering the release of pro-inflammatory cytokines such as IL-17, IL-23 and TNF-α, which drive T helper (Th)1/Th17-mediated inflammation (Horváth et al., 2019; Xie et al., 2023). This model provides a robust platform to investigate the roles of DCs and B cells in psoriasis-like inflammation across key time points: day 3 (early immune activation), day 5 (peak immune infiltration) and day 7 (inflammatory progression). This study examines immunological stages without cross-day comparisons, as separate cohorts at each time point may introduce variability. Instead, trends in DCs and B cells throughout disease progression provide a clearer understanding of psoriasis progression. By analyzing immune dynamics at day 3, day 5 and day 7, this study provides a comprehensive view of the roles of DCs and B cells in disease progression, from early immune activation to sustained inflammation.

IMQ application produced visible lesions with erythema, scaling and increased skinfold thickness (Jabeen et al., 2020; Zhou et al., 2020). These early changes reflected keratinocyte hyperproliferation and immune cell infiltration (Jabeen et al., 2020; Zhou et al., 2020). PASI scoring confirmed a significant increase compared to controls (Van Der Fits et al., 2009; Matza et al., 2019; Bharatha et al., 2024). Histology showed epidermal hyperplasia, hyperkeratosis and dermal immune cell infiltration (Gallegos-Alcalá et al., 2021; Deng et al., 2023). In addition, reduced collagen density and angiogenesis were detected, likely mediated by VEGF and TNF-α (Lu et al., 2021; Chokshi et al., 2023). These observations are consistent with reports that IMQ disrupts keratinocyte differentiation, leading to undifferentiated cell accumulation and psoriatic plaque formation (Choi et al., 2021; Qin, Ma & Qin, 2022).

Early DC activation appears to be a key initiating event in IMQ-induced skin inflammation, as evidenced by increased DC presence and antigen-presentation–related gene expression (Qie et al., 2020; Brunner et al., 2023). This aligns with studies showing that DCs are among the first responders in psoriasis, activated by TLR7 signaling and releasing pro-inflammatory cytokines (Kim et al., 2018; Wang & Bai, 2020). Tortola et al. (2012) and Yoshiki et al. (2014) demonstrated that DC-depleted mice exhibit reduced inflammation, emphasizing the central role of DCs in driving psoriatic pathology. The early elevation of *BAFF* expression further supports B cell survival and differentiation, while increased *CXCR5* suggests the migration of immune cells to inflamed tissues (Niu et al., 2015; Schweighoffer & Tybulewicz, 2021). Singh et al. (2016) reported that monocyte-derived DCs (moDCs) migrate to inflamed tissues, exacerbating psoriasis, which supports our findings of potential early systemic DC mobilization.

As the disease progressed to day 5, the emergence of more severe skin lesions reflected a transition from early inflammation to keratinocyte-driven pathology, characterized by excessive epidermal proliferation and tissue remodeling (Choi et al., 2021; Qin, Ma & Qin, 2022). Histopathological changes at this stage are consistent with sustained immune–keratinocyte crosstalk, leading to disrupted epidermal differentiation and dermal architecture (Qie et al., 2020; Brunner et al., 2023). The involvement of growth factors such as EGF and KGF suggests that growth factor–mediated signaling may amplify keratinocyte hyperproliferation, thereby contributing to lesion expansion and worsening disease severity (Chen et al., 2023).

At this stage, the concurrent accumulation of DCs and B cells in the skin suggests the establishment of a highly activated immune microenvironment, in which antigen presentation, cytokine production and chemokine signaling converge to sustain inflammation (Bošnjak et al., 2022). The upregulation of *H2-Aa*, *BAFF*, *IL-6* and *CXCR5* genes functional coupling between DC activation and B cell recruitment and activation, consistent with findings by Yanaba et al. (2013), who demonstrated the regulatory role of B cells in psoriasis-like inflammation. The decline in circulating B cells suggested their active migration to inflamed skin, guided by chemokines such as CXCL12 and CCL21 (Pan et al., 2022). Chakraborty et al. (2024) and Park et al. (2021) demonstrated the role of chemokines in B cell trafficking, supporting our observations. Additionally, Kim et al. (2018) identified the CD301b^+^ dermal DC subset as a key producer of IL-23, exacerbating early inflammation, which aligns with our findings of sustained DC activation.

At the late stage of disease, the skin exhibited features consistent with fully established psoriatic pathology, characterized by profound epidermal remodeling and sustained immune activation (Nussbaum, Chen & Ogg, 2021; Ortiz-Lopez, Choudhary & Bollag, 2022). Histology reflected chronic keratinocyte dysregulation alongside impaired skin integrity, mimicking human psoriasis (Khaleel, Zalzala & Rashid, 2023; Tran et al., 2024). The coarse texture of the skin, driven by keratinocyte activation and abnormal differentiation, was most evident at this stage (Ortiz-Lopez, Choudhary & Bollag, 2022; Bharatha et al., 2024; Tran et al., 2024). These findings are consistent with reports that IMQ-induced inflammation disrupts keratinocyte differentiation, leading to the accumulation of undifferentiated cells and the formation of psoriatic plaques (Choi et al., 2021; Qin, Ma & Qin, 2022; Xue et al., 2023). Persistent elevation of DCs and B cells, together with sustained expression of *BAFF*, *CXCR5* and *IL-6*, suggests the maintenance of a self-amplifying inflammatory loop, driven by ongoing antigen presentation, cytokine signaling and immune cell recruitment (Kim et al., 2018; Bošnjak et al., 2022). Continued redistribution of B cells from the circulation to inflamed skin is consistent with chemokine-mediated retention within lesional tissue, reinforcing chronic inflammation (Pan et al., 2022). Song et al. (2024) and Sun et al. (2024b) further demonstrated that DCs in psoriatic lesions enhance T cell activation, perpetuating inflammation. Moreover, Fuentes-Duculan et al. (2017) identified autoantigens such as LL-37 and ADAMTSL5 in psoriatic skin, supporting the role of B cells in autoantibody production, which may contribute to chronic inflammation. Collectively, these mechanisms support a model in which DC-B cell interactions and keratinocyte dysregulation converge to drive chronic disease maintenance.

This study has several limitations that should be considered when interpreting the findings. The mechanisms driving the observed cellular and molecular changes were not directly explored and broader immune profiling was not performed. Functional assays to assess DC and B cell activities such as cytokine production, antigen presentation, or cell–cell interactions were not conducted herein. The study focused on three discrete time points, which may miss finer kinetics of immune cell activation and recruitment. Mechanistic insights into signalling pathways, chemokine-mediated recruitment and tissue-specific immune dynamics were not examined. No interventional approaches were applied to evaluate causal relationships or therapeutic potential. Therefore, future studies could address these gaps by implementing functional assays, investigating molecular mechanisms in performing more detailed temporal analyses and testing targeted interventions to confirm the roles of DCs and B cells in psoriasis progression.

## Conclusions

This study demonstrates that progressive immune activation drives IMQ-induced psoriasis-like inflammation, with DCs and B cells actively modulating immune responses beyond their conventional roles. Early CD11c^+^MHCII^+^ DC activation, marked by increased *CD11c* and *H2-Aa* expressions, suggests a role in antigen presentation and cytokine production, amplifying inflammation. The accumulation of CD19^+^CD38^+^ B cells, alongside elevated *BAFF* and *CXCR5* expression, indicates immune crosstalk that facilitates B cell recruitment, activation and migration. Elevated *IL-6* levels indicate ongoing inflammation, while the temporary rise in *IL-10* points to a short-term regulatory response that fades as the inflammatory process continues.

Although T cells are central to psoriasis pathogenesis, DCs and B cells contribute by shaping local inflammation through antigen presentation, cytokine production and potential antibody-independent mechanisms. Understanding their roles is fundamental to unravelling disease persistence and severity. Notably, this study is the first to examine DC and B cell changes in an IMQ-induced psoriasis-like model across multiple time points while incorporating immune profiling of skin samples. This framework captures psoriasis development and the contributions of DCs and B cells in immune regulation in a concise and informative manner.

##  Supplemental Information

10.7717/peerj.20974/supp-1 Supplemental Information 1 Raw data of the obtained results

10.7717/peerj.20974/supp-2 Supplemental Information 2 Supplemental Data Statistical analysis results, including mean ± SD values, median differences (effect sizes), 95% confidence interval (CI) and p-values with their significance indicators for all applicable comparisons across different time points and tissues in the IMQ-induced psoriasis-like mouse model.

10.7717/peerj.20974/supp-3 Supplemental Information 3 Author Checklist for The ARRIVE Guidelines 2.0

## Additional Information and Declarations

### Data Availability

The following information was supplied regarding data availability: The raw data are available in the Supplemental File.
