# Supplementary material for: Role of dendritic cells and B cells in the skin of imiquimod (IMQ)-induced psoriasis-like mouse model
Source: PeerJ. 2026 Mar 30;14:e20974. doi: 10.7717/peerj.20974 (PMC13045838; doi:10.7717/peerj.20974)
Supplement: Supplemental Information 2 — Statistical analysis results, including mean ± SD values, median differences (effect sizes), 95% confidence interval (CI) and p-values with their significance indicators for all applicable comparisons across different time points and tissues in the IMQ-induced psoriasis-like mouse model. [file peerj-14-20974-s002.pdf]

Supplemental Data: Table 1

Parameter	Day	Control (mean ± SD)	IMQ-induced (mean ± SD)	Median Difference	95% CI	*p*-value	Significance
**Skinfold thickness (mm)**							
Day 3 experiment	1	0.517 ± 0.060	0.700 ± 0.050	0.25	-	0.100	NS
	2	0.517 ± 0.060	0.800 ± 0.060	0.30	-	0.009	**
	3	0.533 ± 0.056	0.950 ± 0.070	0.50	-	0.002	**
Day 5 experiment	1	0.417 ± 0.017	0.433 ± 0.021	0.00	-	>0.999	NS
	2	0.417 ± 0.017	0.550 ± 0.022	0.15	-	0.009	**
	3	0.400 ± 0.000	0.917 ± 0.091	0.4	-	0.002	**
	4	0.400 ± 0.000	1.033 ± 0.088	0.55	-	0.002	**
	5	0.400 ± 0.000	1.117 ± 0.111	0.60	-	0.002	**
Day 7 experiment	1	0.500 ± 0.000	0.500 ± 0.000	0.00	-	>0.999	NS
	2	0.500 ± 0.000	0.667 ± 0.062	0.10	-	0.015	*
	3	0.500 ± 0.000	0.700 ± 0.063	0.20	-	0.002	**
	4	0.500 ± 0.000	0.783 ± 0.075	0.30	-	0.002	**
	5	0.500 ± 0.000	1.000 ± 0.103	0.50	-	0.002	**
	6	0.500 ± 0.000	1.100 ± 0.134	0.65	-	0.002	**
	7	0.500 ± 0.000	1.200 ± 0.110	0.75	-	0.002	**
**PASI score**							
Day 3 experiment	1	0.000 ± 0.000	0.000 ± 0.000	0.00	-	0.400	NS
	2	0.000 ± 0.000	2.167 ± 0.167	2.00	-	0.002	**
	3	0.000 ± 0.000	5.333 ± 0.211	5.00	-	0.002	**
Day 5 experiment	1	0.000 ± 0.000	0.000 ± 0.000	0.00	-	0.002	**
	2	0.000 ± 0.000	2.167 ± 0.167	0.20	-	0.002	**
	3	0.000 ± 0.000	5.333 ± 0.211	5.00	-	0.002	**
	4	0.000 ± 0.000	5.333 ± 0.212	5.00	-	0.002	**
	5	0.000 ± 0.000	6.500 ± 0.341	6.00	-	0.002	**
	1	0.000 ± 0.000	0.000 ± 0.000	0.00	-	0.400	NS

Day 7 experiment	2	0.000 ± 0.000	2.167 ± 0.167	2.00	-	0.002	**
	3	0.000 ± 0.000	5.333 ± 0.211	5.00	-	0.002	**
	4	0.000 ± 0.000	5.333 ± 0.212	5.00	-	0.002	**
	5	0.000 ± 0.000	6.500 ± 0.342	6.00	-	0.002	**
	6	0.000 ± 0.000	6.833 ± 0.307	7.00	-	0.002	**
	7	0.000 ± 0.000	7.500 ± 0.548	7.50	-	0.002	**
**Cell population (%)**							
CD11c$^+$MHCII$^+$ DCs	3	0.856 ± 0.618	26.220 ± 7.000	26.83	18.97 – 31.75	<0.0100	**
	5	19.050 ± 4.020	22.470 ± 2.960	2.70	-1.13 – 7.96	0.1250	NS
	7	10.790 ± 1.867	34.970 ± 4.589	25.10	19.67 – 28.68	<0.0001	****
CD19$^+$CD38$^+$ B Cells	3	28.35 ± 22.70	29.05 ± 22.60	0.70	-10.20 – 10.13	0.9943	NS
	5	6.75 ± 1.74	9.85 ± 1.78	3.45	0.83 – 5.37	0.0124	*
	7	14.17 ± 3.53	41.02 ± 9.46	29.55	17.66 – 36.04	<0.0001	****
**Gene expression (relative gene quantification)**							
CD11c	3	0.742 ± 0.445	1.307 ± 0.984	0.27	-0.42 – 1.55	0.2287	NS
	5	0.567 ± 0.216	1.802 ± 0.799	0.88	0.48 – 1.99	0.0044	**
	7	0.567 ± 0.216	2.130 ± 0.980	1.53	0.42 – 2.10	0.0075	**
H2-Aa	3	0.996 ± 0.286	1.557 ± 0.087	0.731	-0.10 – 1.23	0.0895	NS
	5	0.957 ± 0.311	1.864 ± 0.299	0.67	0.05 – 1.87	0.0616	NS
	7	0.713 ± 0.220	2.024 ± 0.378	1.25	0.91 – 1.71	<0.0001	****
BAFF	3	1.046 ± 0.341	1.427 ± 0.628	0.78	-0.27 – 1.03	0.2210	NS
	5	1.189 ± 0.592	1.915 ± 0.775	0.48	-0.16 – 1.61	0.0980	NS
	7	0.819 ± 0.549	2.132 ± 0.948	1.31	0.32 – 2.31	0.0150	*
IL-10	3	0.878 ± 0.649	1.822 ± 0.515	0.92	0.19 – 1.70	0.0190	*
	5	0.976 ± 0.583	2.072 ± 0.871	0.84	0.14 – 2.05	0.0280	*
	7	0.707 ± 0.709	0.468 ± 0.324	0.00	-0.95 – 0.47	0.4710	NS
IL-6	3	0.431 ± 0.314	1.590 ± 0.923	1.14	0.27 – 2.05	0.0155	*
	5	0.544 ± 0.302	1.715 ± 1.149	0.69	-	0.0152	*
	7	0.463 ± 0.370	1.732 ± 0.487	1.28	0.71 – 1.83	0.0005	***
CXCR5	3	0.402 ± 0.767	1.482 ± 1.094	1.08	-	0.0260	*
	5	0.518 ± 0.239	1.475 ± 0.384	0.91	0.55 – 1.37	0.0004	**

	7	0.519 ± 0.257	1.798 ± 0.567	1.35	0.71 – 1.85	0.0005	**

Note: SD = standard deviation, CI = confidence interval, - = not applicable, *$p < 0.05$, **$p < 0.01$, ***$p < 0.001$, ****$p < 0.0001$, NS = not significant
